# Functional connectomes of akinetic‐rigid and tremor within drug‐naïve Parkinson's disease

**DOI:** 10.1111/cns.14284

**Published:** 2023-06-12

**Authors:** Haoting Wu, Cheng Zhou, Xiaojun Guan, Xueqin Bai, Tao Guo, Jingjing Wu, Jingwen Chen, Jiaqi Wen, Chenqing Wu, Zhengye Cao, Xiaocao Liu, Ting Gao, Luyan Gu, Peiyu Huang, Xiaojun Xu, Baorong Zhang, Minming Zhang

**Affiliations:** ^1^ Department of Radiology, The Second Affiliated Hospital Zhejiang University School of Medicine Hangzhou China; ^2^ Department of Neurology, The Second Affiliated Hospital Zhejiang University School of Medicine Hangzhou China

**Keywords:** akinetic‐rigid, connectome, Parkinson's disease, resting‐state fMRI, tremor

## Abstract

**Aims:**

To detect functional connectomes of akinetic‐rigid (AR) and tremor and compare their connection pattern.

**Methods:**

Resting‐state functional MRI data of 78 drug‐naïve PD patients were enrolled to construct connectomes of AR and tremor via connectome‐based predictive modeling (CPM). The connectomes were further validated with 17 drug‐naïve patients to verify their replication.

**Results:**

The connectomes related to AR and tremor were identified via CPM method and successfully validated in the independent set. Additional regional‐based CPM demonstrated neither AR nor tremor could be simplified to functional changes within a single brain region. Computational lesion version of CPM revealed that parietal lobe and limbic system were the most important regions among AR‐related connectome, and motor strip and cerebellum were the most important regions among tremor‐related connectome. Comparing two connectomes found that the patterns of connection between them were largely distinct, with only four overlapped connections identified.

**Conclusion:**

AR and tremor were found to be associated with functional changes in multiple brain regions. Distinct connection patterns of AR‐related and tremor‐related connectomes suggest different neural mechanisms underlying the two symptoms.

## Graphical abstract

The akinetic‐rigid (AR) related and tremor‐related connectomes were detected from drug‐naïve Parkinson disease patients and further verified in an independent set. Comparing two connectomes found that the patterns of connection between them were largely distinct and only four overlapped connections identified. Distinct connection patterns of AR and tremor‐related connectomes reveal different neural mechanisms underlying two symptoms.

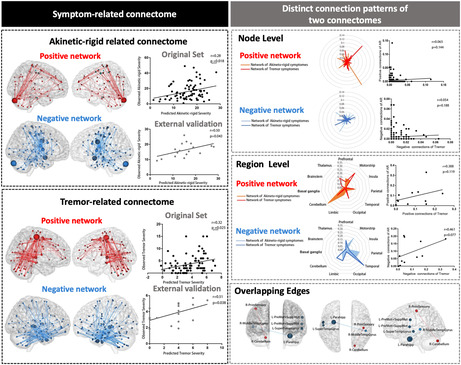

## 1 INTRODUCTION

Parkinson's disease (PD) is the second most common neurodegenerative disease in aging populations, characterized by akinesia, tremor, and rigidity.^1^ Generally, tremor appears as involuntary postural and kinetic movements, and rigidity is clinically associated with the akinetic disorders of the disease, therefore, combined as akinetic‐rigid (AR).^2^ Previous studies had suggested that AR and tremor might have different neural bases since the severity of tremor and AR was not correlated,^3^, ^4^ and patients domained with tremor sometimes follow a more benign disease course than those domained with AR.^5^ Based on these findings, it is crucial to fully understand the respective neural mechanisms underlying AR and tremor so that targeted treatments can be developed for each symptom.

Although significant progress has been made in the understanding of the motor impairment within PD, the detailed pathophysiology of specific symptoms remains largely unknown. Traditionally, motor symptoms were thought to be linked with dysfunction of the motor circuit, which connects the motor cortex to a particular territory within the basal ganglia nuclei.^6^, ^7^ Dopamine deficiency in PD led to increased activity in the indirect circuit, where hyperactivity in the subthalamic nucleus was a key feature and hypoactivity in the direct circuit. As a result, the globus pallidus pars interna/substantia nigra pars reticulata output was elevated, the ventrolateral nucleus of the thalamus was inhibited, and neural activity of motor regions was reduced.^8^, ^9^ However, this perspective is no longer considered adequate to explain motor symptoms of PD.^10^, ^11^ Some researchers have suggested that both AR and tremor may be associated with higher‐order motor functions, such as movement sequencing and execution.^12^, ^13^ There is also evidence that nonmotor factors, such as mental slowness and cognitive loads, could increase the severity of symptoms.^14^, ^15^, ^16^ Accordingly, regions beyond the motor circuit could also contribute to symptoms, and both AR and tremor should be viewed as the result of a malfunction within several connected regions, rather than the outcome of a single circuit failure. Taken together, identifying connectomes related to AR and tremor at the whole‐brain level can improve understanding of their neural basis.

Resting‐state functional MRI (Rs‐fMRI) allows simultaneous investigation of different brain regions and is, therefore, suitable for detecting symptom‐related functional bases across the whole brain.^17^ Several studies performing whole‐brain analyses applying rs‐fMRI have detected functional underpinnings for AR and tremor symptoms beyond the motor circuit at the group level.^18^, ^19^, ^20^ These results, however, are generated at the group level by detecting differences between AR‐dominant subtypes and tremor‐dominant subtypes. In addition to not accounting for the change in subtypes associated with the progression of the disease,^21^ these group‐level analyses do not consider individual differences. As the evidence demonstrated that functional connection patterns are effective for individuals,^22^ the individual variance has been suggested to be informative.^23^ Finding the link between individual behavior and individual connections had been suggested to decrease the bias inherent in population variation, and contribute to more robust and generalized brain‐function association.^17^ Therefore, connectome‐based predictive modeling (CPM), a newly developed data‐driven protocol that allows for one‐to‐one mapping, was applied in the current study for detecting complex connectomes related to symptoms at the individual level.^24^

The effects of the medication should also be taken into account when analyzing the relationship between functional connectomics and motor symptoms. It has been known that dopaminergic medicine regulates the functional activity of the circuits involved in motor processes,^25^ and it could improve motor impairment in PD.^26^ Even though the abovementioned studies were performed off‐state, the long‐term effects of dopamine can also mask the intrinsic neuropathology of symptoms.^27^ By using the CPM method with drug‐naive patients, it would be possible to detect pathophysiological changes related to AR and tremor independent of the reorganization processes induced by medication.

Therefore, this study sought to identify connectomes related to AR and tremor at the individual level among drug‐naive PD patients. The detected connectomes were then validated among an independent set of drug‐naive patients to verify their replication. As mentioned previously, the two symptoms are thought to link with different neural bases, thus connectomes of AR and tremor should be distinct. To verify this hypothesis, we then compare the connection patterns of these two connectomes. In addition, we compared connectome strength between patients and normal controls to determine further clinical relevance.

## 2 MATERIALS AND METHODS

### 2.1 Participants' enrollment and evaluation

A total of 87 drug‐naive patients were initially recruited. All patients provided written informed consent forms following the approval of the Medical Ethics Committee of our institution. Diagnosis of PD was made by a senior neurologist (B.R.Z.) according to the United Kingdom PD Society Brain Bank criteria^28^ and the Movement Disorder Society (MDS) clinical diagnostic criteria of Parkinson's disease.^29^ Six patients were excluded due to cerebrovascular disorders (*N* = 3) and cognitive impairment based on the Mini‐Mental State Examination (MMSE) score (*N* = 3).^30^, ^31^ The remaining 81 patients were followed up at least one time after their first visit. The median follow‐up time was 2.66 years. In the follow‐up, the diagnosis of PD was clinically reevaluated to rule out potential misdiagnosis of atypical parkinsonism. And no patients were excluded during follow‐up.

The severity of symptoms was evaluated according to the Unified Parkinson's Disease Rating Scale part III (UPDRS III) for each patient. The akinetic‐rigid (AR) was evaluated by summarizing the rating on rigidity, finger tapping, hands movements, rapid alternating movements of hands, leg agility, arising from the chair and body bradykinesia, and the tremor was assessed as a sum of rating on resting tremor of the face, arms and legs, and action or postural tremor of the arms.^32^ A higher score indicates increased severity of the symptom.

### 2.2 Image acquisition and preprocessing

#### 2.2.1 Image acquisition

All imaging data, which included Rs‐fMRI and T1‐weighted sequences, were acquired from a 3.0 Tesla MRI scanner (Discovery MR750; GE Healthcare). The head of each participant was stabilized with foam pads, and earplugs were provided to reduce the noise during scanning. The parameters of the two sequences were detailed in Appendix S1.

#### 2.2.2 Image preprocessing

Rs‐fMRI data processing was carried out using Statistical Parametric Mapping version 12 and Data Processing Assistant for Resting‐State fMRI.^33^ In the beginning, the first 10 volumes of the functional time series were deleted letting the MRI signal reach the equilibrium. The remaining images underwent slice timing for interval scanning, realignment, and normalizing to the standard MNI space through T1 images. All data were visually checked after normalizing. Next, spatial smoothing with a Gaussian kernel of 6 × 6 × 6 mm full‐width‐at‐half‐maximum, detrending, covariates regression (Friston 24‐motion parameters, mean signals of white matter and cerebrospinal fluid) and band‐pass temporal filtering (0.01–0.1 Hz) were sequentially applied to the remaining volumes. Considering the effect of head‐motion on the rs‐fMRI analysis, volumes with mean frame‐wise displacement (FD) ≥0.2 mm were removed and the remaining volumes were applied for network construction. Three patients with data shorter than 4 min (120 volumes) after scrubbing were excluded from the following analysis.^34^, ^35^, ^36^ Finally, the rs‐fMRI data of 78 PD patients were enrolled for constructing connection matrices.

#### 2.2.3 Functional connection matrices construction

The whole‐brain functional connection matrix was constructed for each patient in the MNI space. Network nodes were defined using the 268‐ROI functional brain atlas.^37^ The mean time series of each node was extracted by averaging the time courses of all constitute voxels. Node‐by‐node pairwise correlations were then calculated, and Pearson's correlation coefficients (*r*) were Fisher's *r*‐to‐*z* transformation to construct 268 × 268 matrices. Each value of the matrix represented the connection strength between all pairs of nodes.

### 2.3 Symptom‐related connectome construction and evaluation

**FIGURE 1:**
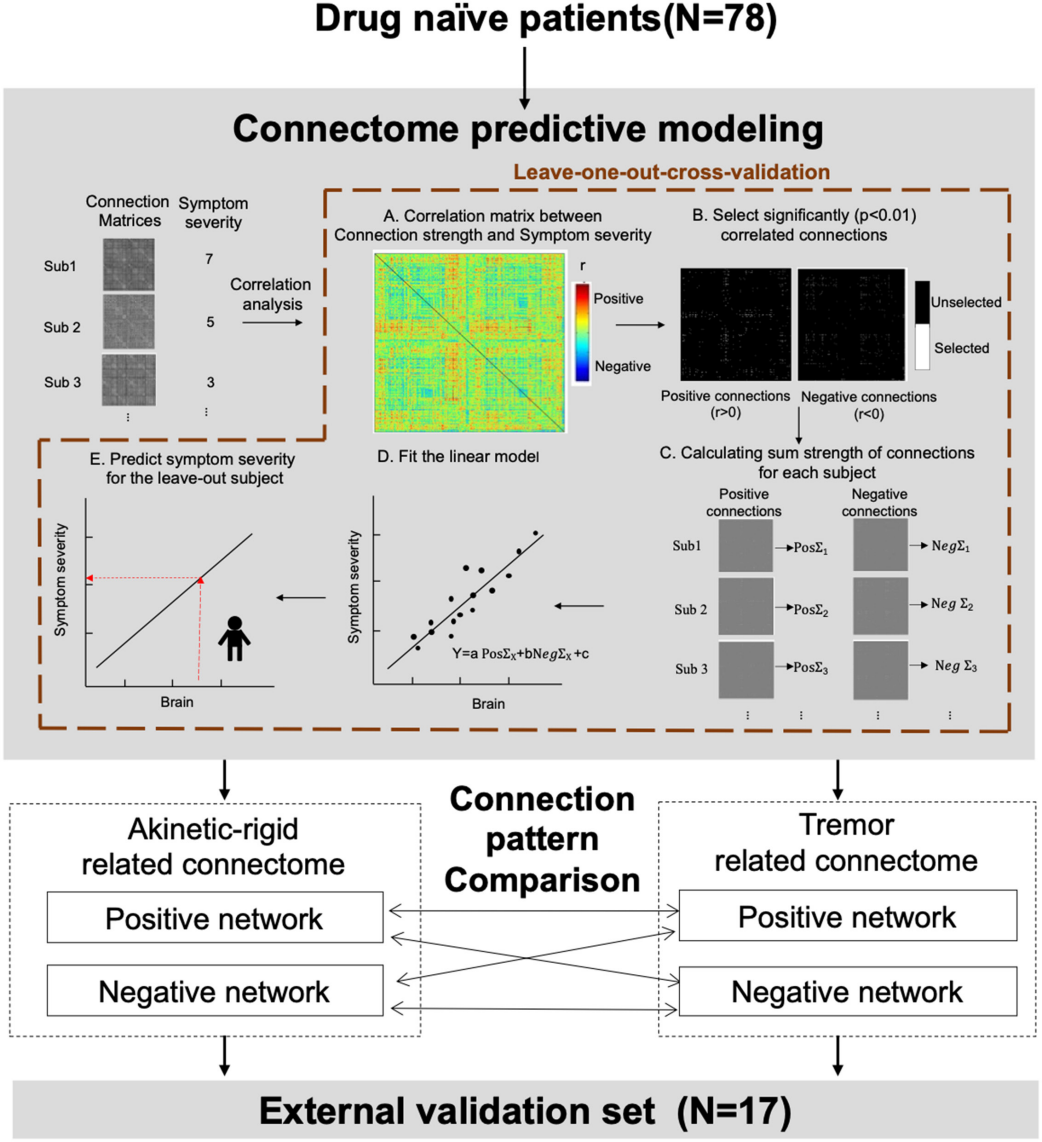
The flowchart of connectomes construction and evaluation. In this study, we employed connectome predictive modeling (CPM) with leave‐one‐out‐cross‐validation (LOOCV) to detect connectome related to akinetic‐rigidity and tremor in 78 drug‐naïve patients (A–E). All selected connections were divided into positive and negative networks based on correlation coefficients of their strength and symptom severity, and then their connection patterns were compared. The detected connectomes were further validated among an external validation set (*N* = 17).

The flowchart of connectomes construction and evaluation was shown in Figure 1.

#### 2.3.1 Symptom‐related connectome constructing via connectome‐based predictive modeling (CPM)

The CPM was used to detect connectomes related to two symptoms. Due to the small number of patients, leave‐one‐out‐cross‐validation was performed as internal validation during model construction.^38^ In each iteration, the data of one patient was left out for testing, and the remaining patients (*N* = 77) were enrolled for training. First, in the training set, the partial correlation analysis (controlling for age, gender, and disease duration) between connection strength and symptoms severity (i.e., AR severity for AR‐related connectome, and tremor severity for tremor‐related connectome) was performed (Figure 1A). Connections with significant correlation coefficients (*p* < 0.01) were selected and divided into positive (*r* > 0) and negative (*r* < 0) connections according to correlation coefficient.^24^ All selected connections were marked and then two binarized “masks” were produced (Figure 1B). Next, the binarized “masks” were returned to the individual connection matrix and the sum strength of negative and positive connections were calculated, respectively (Figure 1C). Subsequently, a model was built by assuming a linear relationship between the sum of the strength of negative and positive connections (independent variables) and symptom severity (dependent variable) (Figure 1D). Finally, the sum strength was calculated for test one (i.e., the leave‐out one) and then input into the linear model to obtain the predicted severity (Figure 1E).

A total of 78 iterations were performed until all patients had been left out at once. To reduce potential variation, only connections shared across every iteration (i.e., common connections) were retained in the connectomes. Each connectome was composed of positive network (comprising positive connections selected across all iterations) and negative network (comprising negative connections selected across all iterations). The network strength was determined by adding up the strength of all connections enrolled in it.

#### 2.3.2 Significance evaluation of detected connectomes

The significance of the detected connectome was quantified through Spearman correlation analysis between the observed and predicted severity because neither AR nor tremor severity was normally distributed. As the cross‐validation analyses of the leave‐out folds are not independent and thus the degree of freedom for parametric *p* values is overestimated, the 1000‐time permutation test was performed to further ensure the significance. In the permutation test, the behavior data and connection matrices were randomly shuffled by permuting patients' labels 1000 times and repeated CPM analysis with the shuffled data. Based on these null distributions, the *p* values of predictabilities were calculated (*p*_permu_), and *p*_permu_ <0.05 was considered statistically significant.^39^

#### 2.3.3 Supplementary analyses for connectome construction

Three supplementary analyses were conducted to determine whether head‐motion, threshold *p*‐value for connection selection and applied atlas could influence the significance of the model (detailed in Appendix S1).

#### 2.3.4 External validation in an independent dataset

To validate the generalizability of the detected connectomes, an independent dataset including 21 drug‐naive patients was enrolled for external validation. The same neurologist diagnosed all patients with PD at their first visit using the same criteria, and all were examined with the same scanner as in the original set. Four patients were excluded because of extensive head‐motion. The remaining 17 patients' Rs‐fMRI data were used to construct connection matrices and for further validation.

### 2.4 Connection pattern analyses of constructed connectomes

#### 2.4.1 Regional‐based and computational lesion prediction

As above mentioned, all connection matrixes were defined using the 268‐ROI (i.e., 268 nodes) functional brain atlas. All nodes were divided into 11 macroscale brain regions based on anatomical labels, including the prefrontal lobe, motor strip, insula, parietal lobe, temporal lobe, occipital, limbic, cerebellum, thalamus, basal ganglia, and brainstem.^37^, ^40^

The following analyses were then conducted. First, to determine whether the underlying brain mechanisms to symptoms could be simplified to functional alternation within a single region, 11 region‐based models were constructed using nodes and connections within a single region. Second, to have a further understanding of the importance of each region for prediction, 11 lesioned models were constructed by excluding the nodes of one region and their corresponding connections. Regions that degrade original performance the most upon exclusion would then be assigned greater importance in the connectomics.^38^ CPM was used to construct both region‐based models and lesioned models as described above. The statistical significance of each region‐based or lesioned model was evaluated by a 1000‐times permutation test and corrected by the FDR correction. The predictive ability of each significant model was then compared with the original one by using Steiger's *z*‐test.^41^

#### 2.4.2 Comparison of connection patterns

The connection patterns between AR and tremor connectomes were compared at the node, region, and connection levels. Comparing was conducted between two negative and two positive networks. Furthermore, a cross‐comparison was conducted between negative and positive networks.

At the node and the region level, Pearson's correlation analysis was applied to determine whether the contributions of nodes/regions to AR‐ and tremor‐related connectomes were similar. Significantly higher correlation coefficients indicate greater similarity between connection patterns.^42^ The contribution of one node or region to the positive/negative network was defined as the percentage of positive/negative connections it has to the total number of connections within the network.

At the connection level, the overlapping connections selected in both connectomes were detected. The significance of overlapping was determined with the hypergeometric cumulative density function in MATLAB: *p* = 1‐hygecdf (x, M, K, n) (detailed in Appendix S1). More overlapping connections indicate greater similarity between connection patterns.

### 2.5 Networks strength relative to normal controls

All NCs were recruited from the community and did not suffer from neurological surgery, head injury, intracranial lesion, cerebrovascular diseases, or other neurological and psychiatric diseases. The strength of NC groups was obtained by applying the identified connectomes to the rs‐fMRI connection matrix of each NC. Considering the heterogeneity of PD, all patients were further classified into AR‐dominant (ARD) and tremor‐dominant (TD) subtypes according to the ratio of mean tremor score to mean AR scores (i.e., ARD: ratio < 0.8, TD: ratio > 1).^32^ And the strength of AR predictive networks was compared between ARDs and NCs, and the strength of TD predictive networks was compared between TDs and NCs.

### 2.6 Statistical analysis

The clinical characteristics of all participants were analyzed by using SPSS software (Version 25). The Kolmogorov–Smirnov test was applied to identify the normal distribution of continuous variables. Continuous variables with normal distribution were presented as average ± standard deviation and compared with a two‐sample *t*‐test. Continuous variables with non‐normal distribution were presented as median and range and compared with the Mann–Whitney test. Differences between qualitative variables were compared with the Chi‐square test. A two‐sided *p*‐value <0.05 was considered significant unless mentioned.

Statistical analyses of connectomes construction and evaluation had been detailed in the corresponding parts mentioned above. All processes were performed in MATLAB (R2020b, MathWorks) with custom scripts.^24^ The scripts can be available at https://www.nitrc.org/projects/bioimagesuite/.

## 3 RESULTS

### 3.1 Significant correlation between detected connectome and symptom severity

**FIGURE 2:**
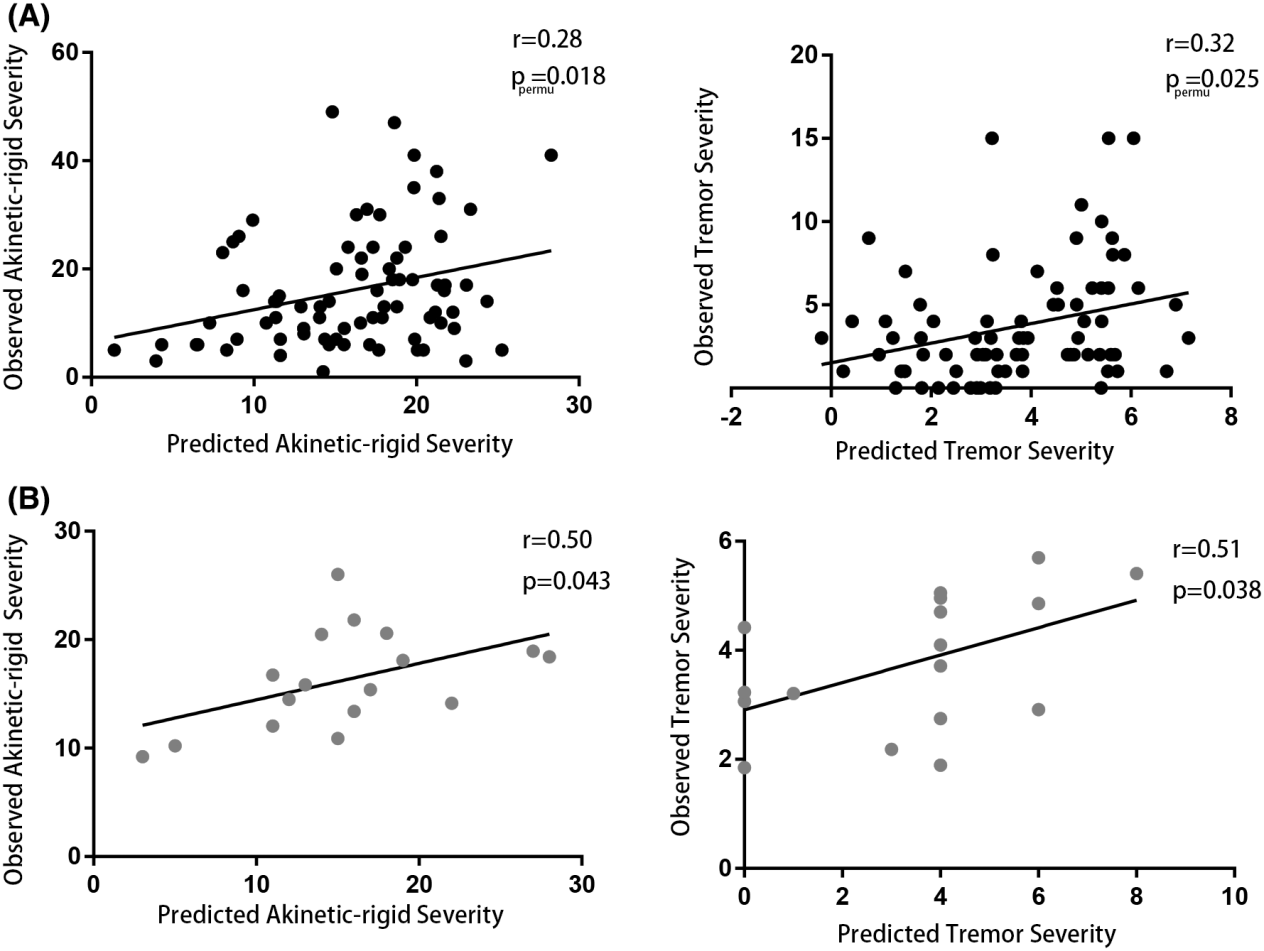
Significant correlation between detected connectome and symptom severity. Spearman correlation analysis demonstrated a significant correlation between predicted and observed akinetic‐rigid and tremor severity in both the original set (A) and external validation set (B). *p*_permu_, The significance of the correlation was evaluated by a 1000‐times permutation test.

Characteristics of enrolled 78 drug‐naïve patients were detailed in Table 1. The median disease duration was 1.64 years, and most patients were in the mild stage (median: HY stage 2).CPM with LOOCV successfully identified connectomes related to AR (*r* = 0.28, *p*_permu_ = 0.018) and tremor (*r* = 0.32, *p*_permu_ = 0.025) (Figure 2A). There was no significant correlation between actual scores and head‐motion (AR: *r* = −0.078, *p* = 0.498; Tremor: *r* = −0.042, *p* = 0.714) as well as predicted scores and head‐motion (AR: *r* = −0.206, *p* = 0.071; Tremor: *r* = −0.128, *p* = 0.263).

**TABLE 1:** Characteristics of enrolled drug‐naïve patients.

Characteristics	Total (*N* = 95)	Original set (*N* = 78)	External validation set (*N* = 17)	*p*
Age (years)	58.05 ± 10.22	57.75 ± 10.83	59.43 ± 6.79	0.540
Gender (M/F)	51/44	44/34	7/10	0.254
MMSE	27 (18–30)	27 (20–30)	25 (18–30)	0.052
Education (years)	9 (0–22)	9 (0–22)	6 (0–12)	0.028*
Disease Duration (years)	1.60 (0.01–7.92)	1.64 (0.01–7.92)	1.51 (0.24–3.88)	0.374
UPDRS III	20 (4–68)	19 (4–68)	23 (7–41)	0.638
Akinetic‐rigid severity	14 (1–49)	13 (1–49)	15 (3–28)	0.610
Tremor severity	3 (0–15)	3 (0–15)	4 (0–8)	0.758
Hoehn and Yahr stage	2 (1–3)	2 (1–3)	2 (1–3)	0.879

*Note*: According to the Kolmogorov–Smirnov test, variables (i.e., age) with normal distribution were presented as average ± standard deviation and compared with a two‐sample *t*‐test, and variables (education, MMSE, disease duration, UPDRS III, tremor severity, akinetic‐rigid severity, and H‐Y stage) with non‐normal distribution were presented as median (range) and compared with Mann–Whitney test. The qualitative variable (i.e., gender) was compared with the Chi‐square test. *p < 0.05.

Abbreviations: MMSE, Mini‐Mental State Examination; UPDRS III, The Unified Parkinson's Disease Rating Scale part III.

Models conducted in supplementary analyses can also significantly predict AR (*r*_max_ = 0.31, *p*_permu_ <0.038) and tremor (*r*_max_ = 0.43, *p*_permu_ <0.018), demonstrating that head‐motion, threshold, or atlas did not influence the significance of prediction (Table S1).

### 3.2 Replication of the detected connectomes in the external validation set

Characteristics of 17 drug‐naïve patients in the external validation set were detailed in Table 1. There were no significant differences between the original set and the external validation set. The predicted severity of each patient was generated via detected connectomes, and Spearman correlation analysis determined the significant correlation between observed and predict AR severity (*r* = 0.50, *p* = 0.043) as well as observed and predict tremor severity (*r* = 0.51, *p* = 0.038; Figure 2B).

### 3.3 Regional‐based and computational lesion prediction

None of the region‐based models could significantly predict two symptoms after the FDR correction (Table S2). Results of lesioned‐model analysis showed that models cannot predict the severity of AR after excluding the parietal lobe (*r* = 0.23, *p* = 0.082, *q* = 0.082) and limbic system (*r* = 0.21, *p* = 0.066, *q* = 0.073) while removing other regions did not significantly decrease the prediction of AR (Table S3A). As for tremor, all lesioned models remained significant prediction after FDR correction, and the Steiger's *z*‐test demonstrated that the prediction of tremor significantly decreased after removing the motor strip (0.32 vs. 0.26, *z* = 2.63, *p* = 0.009) and cerebellum (0.32 vs. 0.27, *z* = 2.03, *p* = 0.043) (Table S3B).

### 3.4 Two connectomes have distinct connection pattern

**FIGURE 3:**
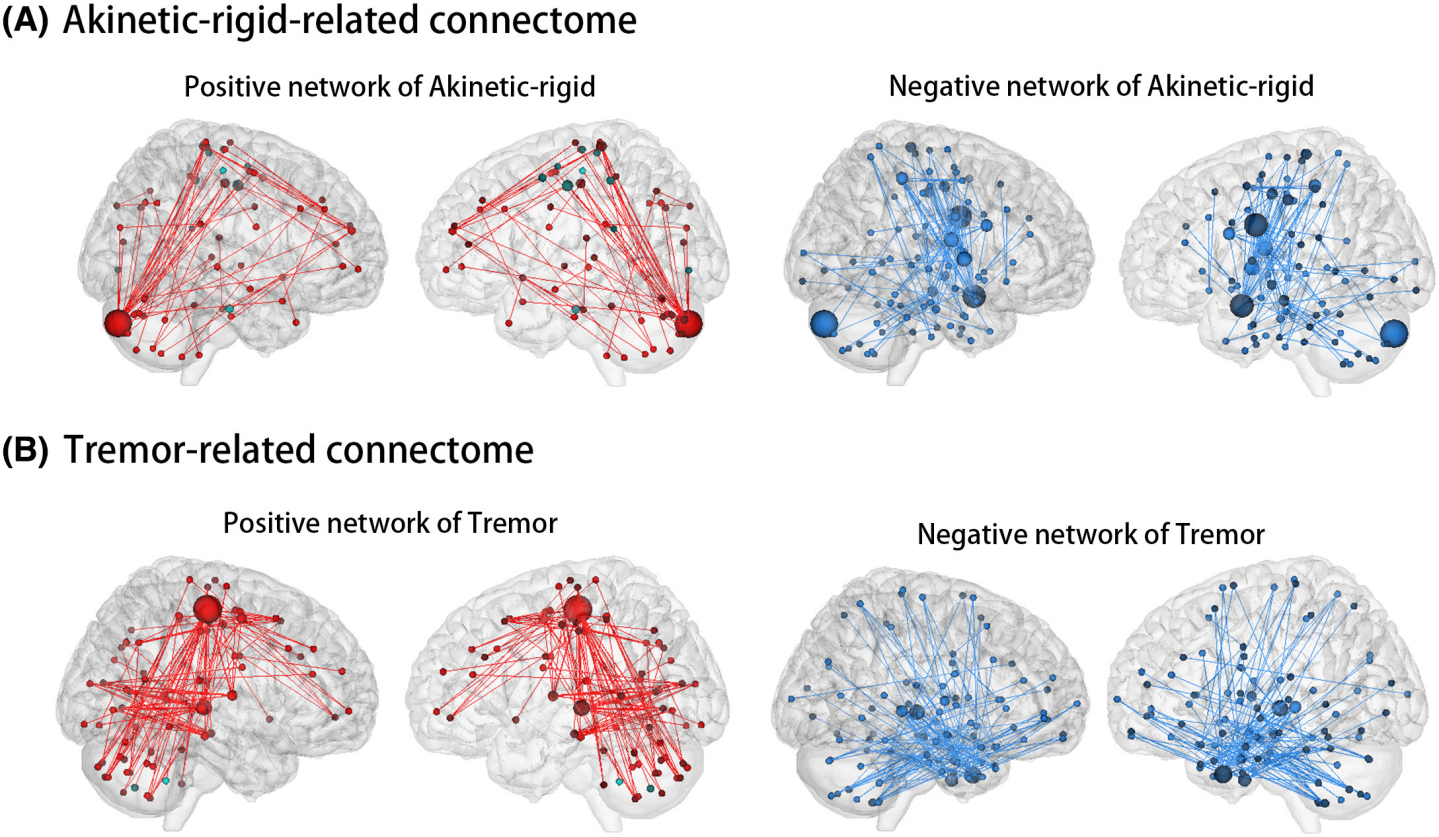
Connection patterns of Akinetic‐rigid‐related connectome (A) and tremor‐related connectome (B). Negative connections were represented by the blue line and positive connections by the red line. Nodes were sized according to the number of connections they contain. A larger node contained more connections.

**FIGURE 4:**
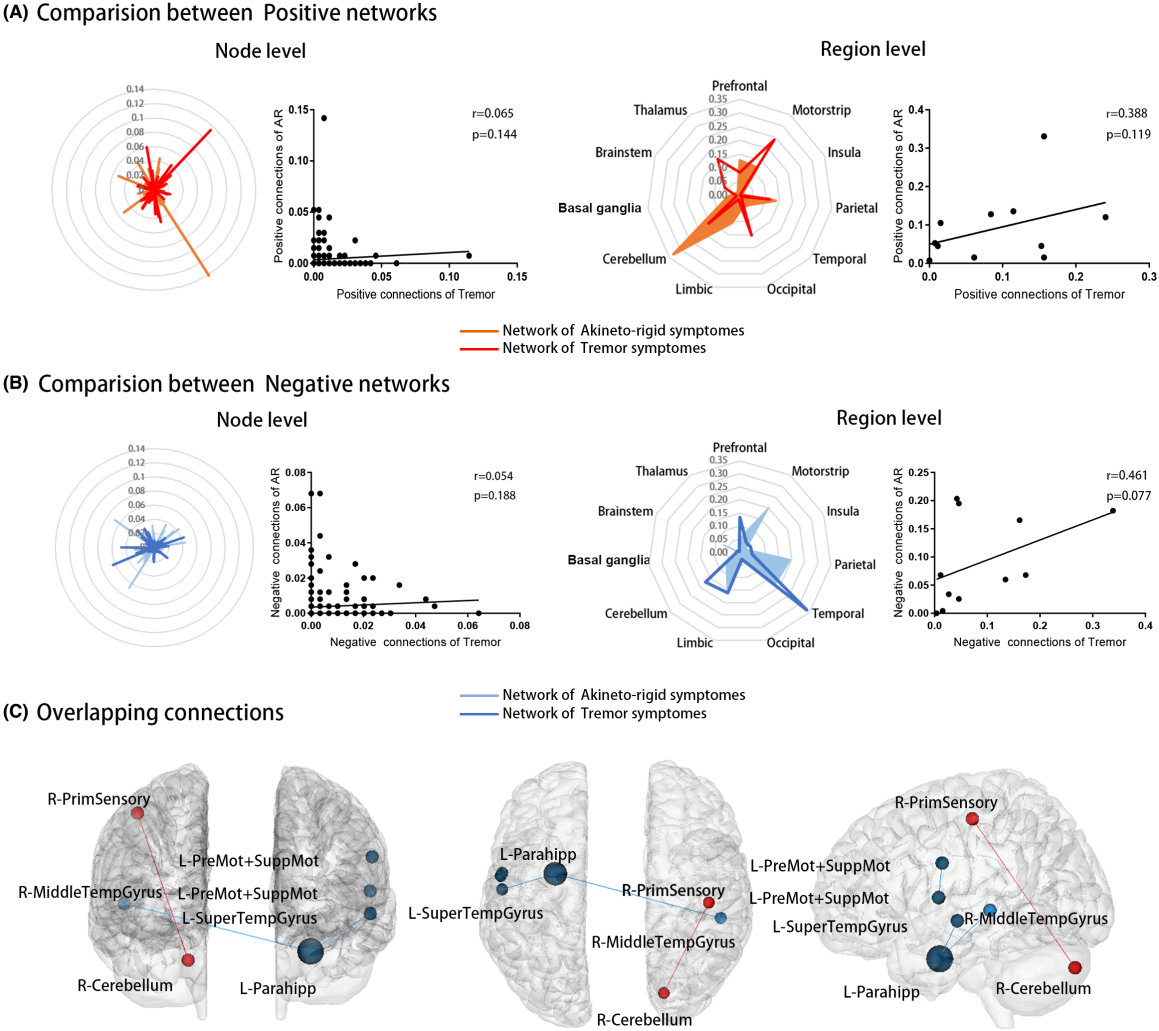
Comparing connection patterns of positive (A) and negative (B) networks at node and region level. Each dot presented the contribution of each node or region to the predictive network. Only four overlapping connections were detected (C), including three negative connections (blue) and one positive connection (red). AR, akinetic‐rigid.

Figure 3 displayed the connection patterns of AR‐ and tremor‐related connectomes. A total of 192 connections were selected for the AR‐related connectome, including 67 positive and 125 negative connections. There were 279 connections in the tremor‐related connectome, including 131 positive connections and 148 negative connections. Figure 4 illustrated the contribution of each node and region to predictive networks. Cerebellum had the most positive connections of AR‐related connectome (*N* = 44), and motor strip had the most positive connections of tremor‐related connectome (*N* = 63). The most negative connections of the AR‐related connectome come from the motor strip (*N* = 48), and the most negative connections of the tremor‐related connectome come from the temporal lobe (*N* = 90). Nodes with a sum of connections ranked in the top 5% (*N* = 12) were listed in Table S4.

The AR and tremor‐positive networks, as well as the AR and tremor negative networks, showed no significant association at the node or region level. There were almost no overlaps between AR and tremor networks, with only four connections (including one positive and three negative connections, *p* = 0.001) between seven nodes overlapped (Figure 4C). The location of overlapped nodes was detailed in Table S5.

The cross‐comparison analysis of networks' connection patterns found a weak correlation between AR‐negative network and tremor‐positive network only at the node level (*r* = 0.11, *p* = 0.042). There was no significant correlation between AR positive network and the tremor negative network at either level. No common connections were detected in the cross‐comparison analysis.

### 3.5 Decreased negative network strength and comparable positive network strength compared to NCs

Characteristics of ARDs, TDs, and NCs were detailed in Table 2. The age, gender, education, and MMSE scores of NCs were not significantly different from ARDs and TDs. ARDs had more severe AR (ARD vs. TD = 14 vs. 9, *p* = 0.008) and milder tremor (ARD vs. TD = 2 vs. 5, *p* < 0.001) than TDs, and no other characteristics were significantly different.

**TABLE 2:** Characteristics comparison between motor subtypes and normal control.

Characteristics	ARD (*N* = 39)	TD (*N* = 33)	NC (*N* = 57)	*p* ARD vs. NC	*p* TD vs. NC	*p* ARD vs. TD
Age (years)	57.38 (34.09–73.66)	56.98 (33.80–33.30)	58.99 (46.91–77.11)	0.371	0.325	0.950
Gender (M/F)	23/16	18/15	23/34	0.073	0.173	0.705
Education (years)	8 (0–22)	9 (0–17)	9 (2–16)	0.259	0.986	0.421
MMSE	27 (21–30)	27 (20–30)	28 (21–29)	0.344	0.645	0.620
Disease Duration (years)	1.39 (0.08–4.03)	1.98 (0.01–7.92)	–	–	–	0.527
UPDRS III	20 (4–68)	15 (5–55)	–	–	–	0.258
Tremor severity	2 (0–10)	5 (1–15)	–	–	–	<0.001*
Akinetic‐rigid severity	14 (3–49)	9 (1–33)	–	–	–	0.008*
Hoehn and Yahr stage	2 (1–3)	2 (1–3)	–	–	–	0.828

*Note*: *p* < 0.05 was considered statistically significant (annotated with *). All continuous variables were in non‐normal distribution. And they were presented as median (range) and compared by the Mann–Whitney test.

Abbreviations: ARD, akinetic‐rigid dominant; H–Y stage, Hoehn and Yahr stage; MMSE, Mini‐Mental State Examination; NC, normal control; TD, tremor dominant; UPDRS III, The Unified Parkinson's Disease Rating Scale part III.

**FIGURE 5:**
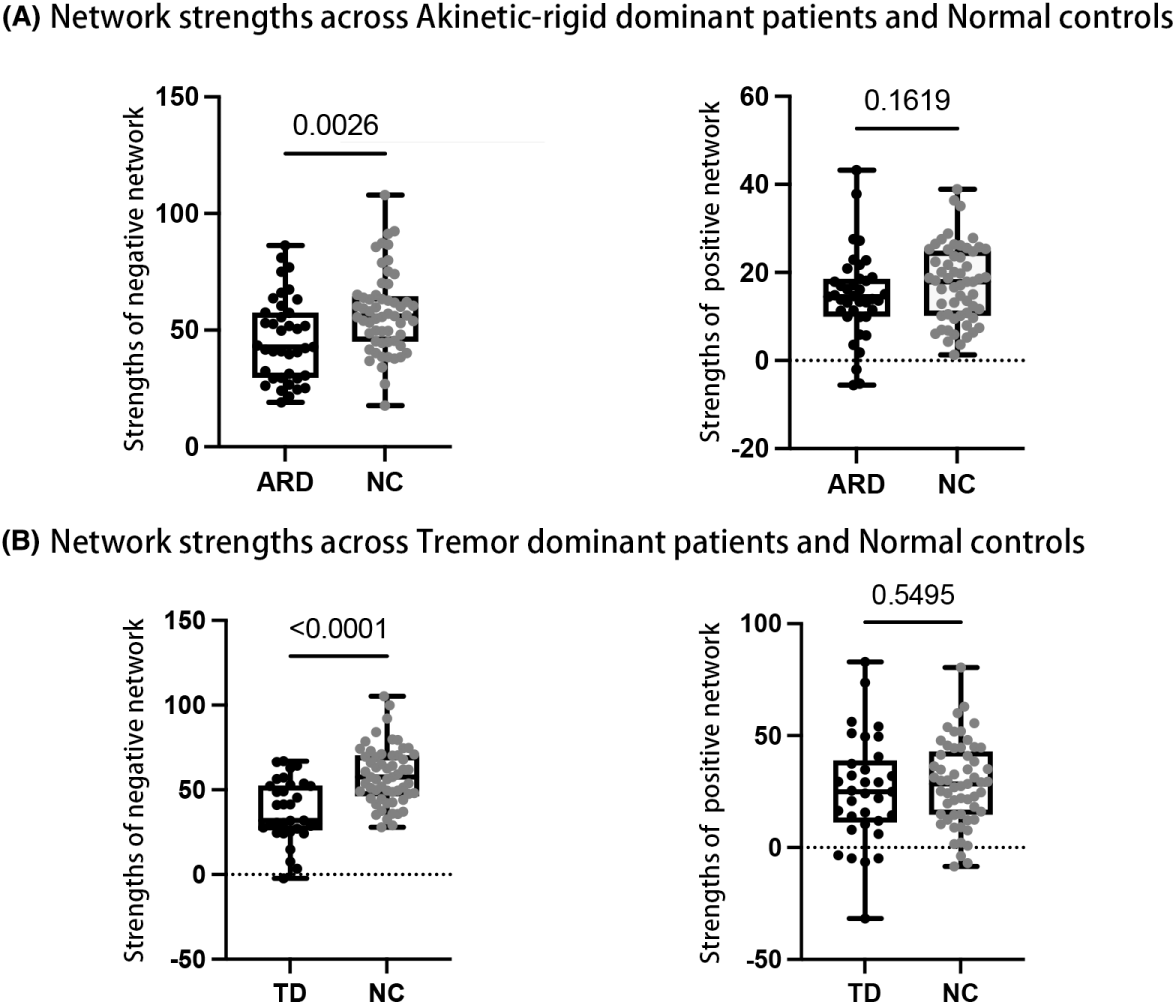
Networks strength across patients and normal controls. ARD, akinetic‐rigid dominant; NC, normal control; TD, tremor dominant.

For the predictive network of AR, results demonstrated that ARDs had decreased negative network strength (ARD vs. NC = 46.22 vs. 57.45, *p* = 0.003) but comparable positive network strength (ARD vs. NC = 14.68 vs. 17.36, *p* = 0.162) relative to NCs (Figure 5A). The same results were also detected in the predictive network of tremor when comparing the network strength of TDs and NCs (negative network: TD vs. NC = 37.62 vs. 58.48, *p* < 0.001; positive network: TD vs. NC = 25.59 vs. 28.32, *p* = 0.550; Figure 5B).

## 4 DISCUSSION

In this study, we identified connectomes of AR and tremor within drug‐naive patients by using CPM. The significant correlation between connectomes and severity was further verified in an independent set. AR and tremor were found to be associated with functional changes in multiple brain regions, neither of which could be simplified to changes within a single region. Results of a computational lesion version of CPM revealed that the parietal lobe and limbic system were the most important regions among AR‐related connectome, and the motor strip and cerebellum were the most important regions among tremor‐related connectome. As we hypothesized, connection patterns of AR‐related and tremor‐related connectomes were different. Additional analysis determined that patients had decreased negative network strength and comparable positive network strength relative to NCs for both symptoms. These two connectomes help to get a full understanding of the neural mechanism specific to AR and tremor and might be targeted in the development of novel therapies.

The parietal lobe and limbic systems were demonstrated to be the most important region for predicting AR severity. Both regions contributed greatly to the negative network whose decreased strength correlated with progressed AR severity. In the parietal lobe, the primary sensory area contained the most negative connections. This area is the central part of sensorimotor integration,^43^ and previous findings had also reported an association between impaired sensory integration and AR.^14^, ^44^ As for the limbic system, the left amygdala contained the most negative connections. The amygdala has long been known to play a significant role in emotional processing aspects,^45^ and recent studies further detected that it might be a key line of the limbic‐motor interface.^46^ The correlation detected in this study might suggest that AR progression could associate with the disordered coordination of emotion and motor in the amygdala. Together, these findings indicated a complex neural basis for AR that can be viewed as a defective integration of a number of motor processes that involve the communication between sensory and emotional signals.

It was not surprising that connections that belong to the motor strip and cerebellum were the most influential for predicting tremor severity, given that both regions were critical parts of tremor circuit.^47^ Specifically, the activity of the motor strip correlated with tremor amplitude, and the activity of the cerebellar associated with tremor rhythm in PD.^48^ The motor strip and cerebellum were found to have the most positive connections in the present study, which showed that enhanced function of these areas was associated with progressive tremor severity. The significant positive relationship between motor strip activity and tremor was consistent with the study applying other functional imaging modalities.^49^ For the cerebellum, a link between tremor suppression and regional cerebral blood flow decline had been detected previously.^50^ These results were consistent with the theory that hyperactivity of the motor strip and cerebellum was crucial to the genesis of tremor.

Comparing connections patterns revealed that nodes or regions contributed differently to AR and tremor negative networks and only three connections overlapped. Most of the connections in the negative network of AR come from cortical regions, mostly motor strip, which was consistent with previous studies demonstrating a correlation between motor strip dysfunction and worsening AR.^44^, ^51^ As for the tremor negative network, most were located in the temporal lobe, while connections from other cortical regions were less prevalent. In previous studies, it was found that patients domained with tremors had lower neural activity in the temporal lobe as compared to other patients.^52^ Furthermore, it has been demonstrated that worsen tremor severity of PD patients was correlated with greater cortical atrophy in the temporal lobe.^53^ It may be possible that tremor may also be related to high‐level functions controlled by the temporal lobe, which could explain to some extent why cognitive or emotional load may exacerbate the condition.^54^ Yet, this inference requires further proof. Based on these findings, both symptoms were correlated with dysfunction in the cerebral cortex with AR‐related dysfunction covering more regions than tremor.

The connection patterns of two positive networks were also distinct, with only one overlapped positive connection detected. As previously mentioned, the majority of the connections in the tremor‐positive network belonged to the motor strip and the cerebellum. The thalamus, another important component of the tremor circuit, also plays an important role in positive networks. In the AR‐related connectomes, most of the positive connections originated from the cerebellum. Researchers had previously observed that PD patients' cerebellar activation increased during motor activities.^55^, ^56^, ^57^, ^58^ Besides, patients dominated with AR were reported to have increased neural activity in the cerebellum in the rest state.^59^ It was unclear what causes the hyperactivity or strengthened connectivity in the cerebellum in PD. Considering the connection pattern of the negative network relating to the AR, we inferred that the increased functional activity of the cerebellum might assist in overcoming the dysfunction of the cerebral cortex.

To determine the further clinical relevance of predictive networks, we compared the network strength with NCs. Results showed that patients had decreased negative network strength and comparable positive network strength relative to NCs for both symptoms. It was possible to infer from these results that negative networks might be indicative of the disease‐related dysfunction of symptoms where some regions might function below normal levels, while positive networks might indicate compensatory mechanisms where some regions might be upregulated to normal levels for a compensatory effect. Combined with the results of the connection patterns comparison, it could interpret that AR and tremor enabled distinct dysfunction and compensation mechanisms. Further cross‐comparisons revealed a weak correlation between the AR‐negative network and the tremor‐positive network. There was a similar contribution from some nodes to the AR‐negative network and tremor‐positive network, according to these findings. It was inferred that tremors might generate to compensate for AR,^2^ although the further analysis was needed, current findings suggested that there may be a correlation between AR‐related dysfunction and tremor‐related compensation.

This study had the following limitations. First, the diagnosis of PD was based on history and physical examination based on clinical criteria. Despite the majority of patients having follow‐up after first visiting to reach an unequivocal diagnosis, combining other examinations, especially dopamine transporter single‐photon emission computed tomography scans, might help to differentiate PD from other parkinsonian syndromes. Second, both symptoms were evaluated within UPDRS scales. Even though this approach is commonly used in clinical practice, it may not be accurate for patients who have mild symptoms due to floor effects. Consequently, validating the connectomes for other quantitative relative tests can further demonstrate their robustness. Third, the connectomes were identified in patients at a relatively early stage of the disease, so further validation with patients at an advanced stage is required. Fourth, results in this study demonstrated different functional connectomes associated with AR and tremor, further studies may add structural data (e.g., diffusion tensor imaging) to provide additional information on the underlying neural base. The primary findings detected in this study need to be validated with external data sets from other institutions.

In conclusion, both AR and tremor should be viewed as connectome dysfunction. In addition to regions included in motor circuits, there were also regions controlling sensory, emotional, and cognitive processes that correlated with symptom severity. Connectomes related to two symptoms had distinct connection patterns, which to some extent reflect their different neural mechanisms.

## AUTHOR CONTRIBUTIONS

H.T.W., C.Z., and X.J.G. contributed to the conception and design of the study. H.T.W., Z.Y.C., X.Q.B., T.G., J.J.W., J.W.C., J.Q.W., C.Q.W., X.C.L., T.G., G.L.Y., and B.R.Z. contributed to the acquisition and analysis of data. H.T.W,C.Z., and X.JG contributed to manuscript drafting. Interpretation of the results, critical revision of the manuscript, and final version approval: all authors.

## CONFLICT OF INTEREST STATEMENT

The authors have declared that no conflict of interest statement.

## Supporting information


Appendix S1

## Data Availability

The materials used and/or analyzed during the current study are available from the corresponding author upon reasonable request.
