# Supplementary material for: Functional connectomes of akinetic‐rigid and tremor within drug‐naïve Parkinson's disease
Source: CNS Neurosci Ther. 2023 Jun 12;29(11):3507–17. doi: 10.1111/cns.14284 (PMC10580330; doi:10.1111/cns.14284)
Supplement: Supplementary file 1 — Appendix S1 [file CNS-29-3507-s001.docx]

Supplementary Materials

Supplementary analyses


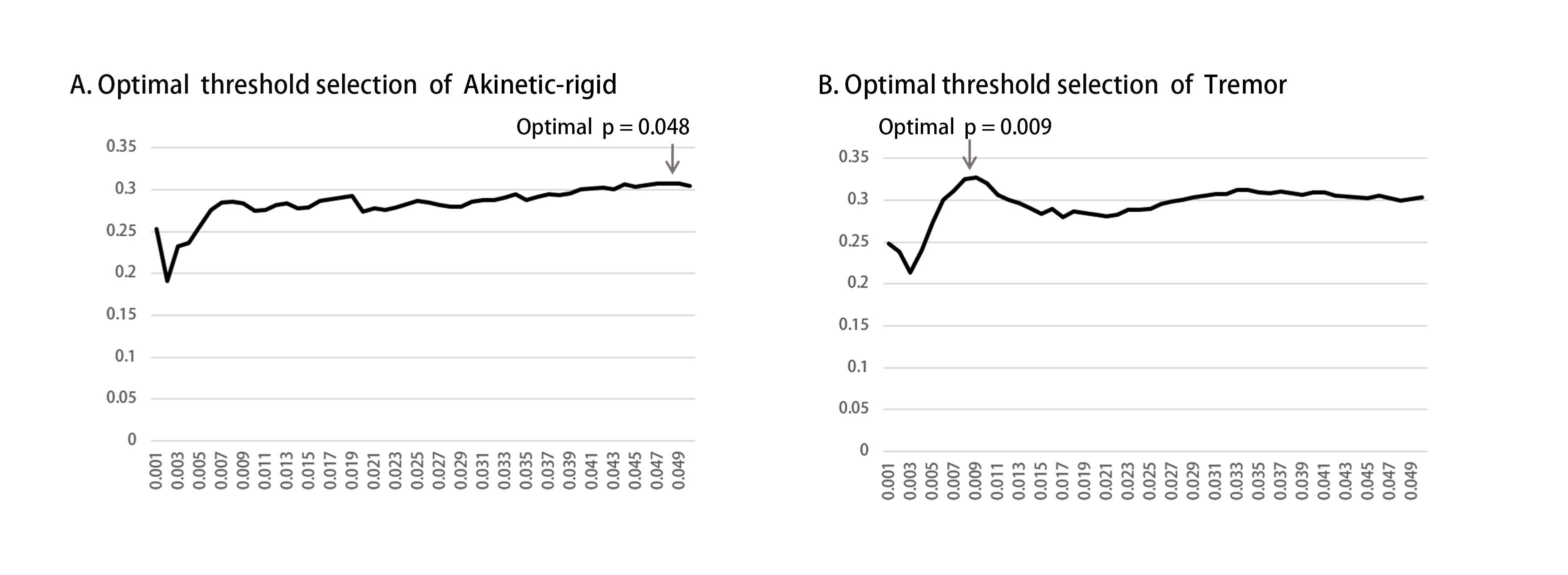
 To determine whether head-motion, threshold p-value for connection selection, and applied atlas could influence the significance of predictions, three supplementary analyses were conducted. First, the influence of head-motion was explored by applying additional prediction analysis with the mean FD as an additional nuisance variable in the CPM process (labeled as AR-M1, Tremor-M1). Second, the effect of the p threshold for connection selection was also examined. By testing a range of p values from 0.001 to 0.05 with an interval of 0.001, the optimal p thresholds that led to the best predictability were obtained for the model of AR (p=0.009, labeled as AR-M2) and the model of tremor (p=0.048, labeled as Tremor-M2) (detailed in the figure below). Third, the Anatomical Automatic Labeling (AAL), which includes 116 regions, was applied to construct connection matrices to explore the influence of atla (labeled as AR-M3, Tremor-M3).

Figure: Optimal threshold selection. The optimal threshold was selected by testing a range of p values from 0.001 to 0.05 with an interval of 0.001. The threshold with a p-value of 0.009 led to the best prediction of AR, and the p-value of 0.048 led to the best prediction of tremor.

Significance of overlapped connections

Overlapping connections that were commonly selected with each iteration to predict AR and tremor were detected. And significance was determined with the hypergeometric cumulative density function in MATLAB, which returns the probability of drawing up to x of K possible items in n drawings without replacement from an M-item population. This was performed as follows: p=1-hygecdf (x, M, K,n) where x equals the number of overlapping connections, n equals the total connections in the first predictive network, K equals the total connections in the second predictive network, and M equals the total number of possible connection the brain (35778).

Parameters of two sequences

RS-fMRI data were acquired using Gradient Recalled Echo - Echo Planar Imaging sequence: TE = 30 ms; TR = 2,000 ms; FA = 77 degrees; FOV = 240 × 240 mm^2^; matrix = 64 × 64; slice thickness = 4 mm; slice gap = 0 mm; number of slices = 38 (axial); time points = 205. Structural T1-weighted images were acquired using a Fast-Spoiled Gradient Recalled sequence: echo time = 3.036 ms; repetition time = 7.336 ms; inversion time = 450 ms; flip angle = 11 degrees; field of view = 260 × 260 mm^2^; matrix = 256 × 256; slice thickness = 1.2 mm; number of slices = 196 (sagittal). All the sequence FOVs covered the whole brain, including cerebrum, cerebellum, and brain stem.

Table S1 Prediction of models constructed in supplementary analyses

| Models | r | P_permu_ |
| --- | --- | --- |
| AR-M1 | 0.29 | 0.020 |
| AR-M2 | 0.31 | 0.006 |
| AR-M3 | 0.29 | 0.038 |
| Tremor-M1 | 0.33 | 0.018 |
| Tremor-M2 | 0.33 | 0.003 |
| Tremor-M3 | 0.43 | 0.003 |

Note: The significance of each model was evaluated by a 1000-times permutation test.AR: akinetic-rigid

TableS2: Prediction of region-based models

A. Model of akinetic-rigid

| Regions | r | p_permu_ | q |
| --- | --- | --- | --- |
| Prefrontal | -0.98 | 0.989 | 0.989 |
| Motor strip | 0.24 | 0.083 | 0.304 |
| Insula | -1.00 | 0.464 | 0.598 |
| Parietal | 0.35 | 0.010 | 0.083 |
| Temporal | 0.20 | 0.116 | 0.319 |
| Occipital | -0.35 | 0.489 | 0.598 |
| Limbic | 0.01 | 0.441 | 0.598 |
| Cerebellum | -0.25 | 0.745 | 0.820 |
| Basal ganglia | -0.86 | 0.288 | 0.598 |
| Brainstem | 0.29 | 0.015 | 0.083 |
| Thalamus | -1.00 | 0.407 | 0.598 |

B. Model of tremor

| Regions | r | p_permu_ | q |
| --- | --- | --- | --- |
| Prefrontal | 0.15 | 0.202 | 0.741 |
| Motor-strip | -0.05 | 0.380 | 0.775 |
| Insula | -1.00 | 0.364 | 0.775 |
| Parietal | -0.85 | 0.817 | 0.817 |
| Temporal | 0.27 | 0.047 | 0.259 |
| Occipital | -0.50 | 0.493 | 0.775 |
| Limbic | -0.27 | 0.758 | 0.817 |
| Cerebellum | -0.14 | 0.634 | 0.775 |
| Basal ganglia | -1.00 | 0.566 | 0.775 |
| Brainstem | -1.00 | 0.566 | 0.775 |
| Thalamus | 0.16 | 0.030 | 0.259 |

Note: The significance of region-based models was evaluated by a 1000-times permutation test combined with FDR correction. While no region-based model remained significant after FDR correction, we did not compare their predictions with the original model.

TableS3Prediction of lesioned models

A. Lesioned models of akinetic-rigid

| Lesioned Regions | r | p_permu_ | q | Z,p | |
| --- | --- | --- | --- | --- | --- |
| -Prefrontal | 0.30 | 0.018 | 0.044 | | 1.080,0.280 |
| -Motor-strip | 0.28 | 0.036 | 0.044 | | 0.170,0.865 |
| -Insula | 0.27 | 0.028 | 0.044 | | 0.136,0.892 |
| -Parietal | 0.21 | 0.082 | 0.082 | | - |
| -Temporal | 0.28 | 0.034 | 0.044 | | 0.132,0.895 |
| -Occipital | 0.31 | 0.020 | 0.044 | | 2.434,0.015 |
| -Limbic | 0.23 | 0.066 | 0.073 | | - |
| -Cerebellum | 0.26 | 0.014 | 0.044 | | 0.213,0.831 |
| - Basal ganglia | 0.28 | 0.025 | 0.044 | | 0.233,0.816 |
| -Brainstem | 0.27 | 0.030 | 0.044 | | 0.445,0.656 |
| -Thalamus | 0.29 | 0.025 | 0.044 | | 1.515,0.130 |

B. Lesioned models of tremor

| Lesioned Regions | r | p_permu_ | q | | Z,p |
| --- | --- | --- | --- | --- | --- |
| -Prefrontal | 0.30 | 0.025 | 0.034 | 1.314,0.189 | |
| -Motor-strip | 0.26 | 0.044 | 0.044 | 2.626,0.009 | |
| -Insula | 0.32 | 0.015 | 0.033 | 0.102,0.919 | |
| -Parietal | 0.32 | 0.021 | 0.033 | 0.309,0.757 | |
| -Temporal | 0.28 | 0.042 | 0.044 | 0.954,0.340 | |
| -Occipital | 0.35 | 0.012 | 0.033 | 1.373,0.170 | |
| -Limbic | 0.31 | 0.019 | 0.033 | 0.474,0.636 | |
| -Cerebellum | 0.27 | 0.035 | 0.043 | 2.028,0.043 | |
| - Basal ganglia | 0.32 | 0.015 | 0.033 | 1.253,0.064 | |
| -Brainstem | 0.33 | 0.014 | 0.033 | 0.274,0.787 | |
| -Thalamus | 0.33 | 0.016 | 0.033 | 0.574,0.566 | |

Note: The significance of each region-specific model was evaluated by a 1000-times permutation test and corrected with FDR. The prediction of each significant model was then compared with the original one by using Steiger’s z test. A). Results showed that after “lesioning” the parietal lobe and limbic system, the akinetic-rigid did not remain significant, and Steiger's z test showed that no lesioned model had significantly decreased prediction relative to the original one. B) Results showed that all “lesioned” models remained with significant predictability for tremor, and Steiger's z test showed that the prediction of the original model significantly decreased after excluding motor strip and cerebellum

TableS4：TOP 5% highest- Degree nodes of different networks

A. Positive network of akinetic-rigid

| Node Number | Sum of connections | Region | Brodmann's area | MNI |
| --- | --- | --- | --- | --- |
| 111 | 19 | R-Cerebellum | Cerebellum | 11.27, -84.31, -34.65 |
| 174 | 7 | L- Parietal | VisMotor | -7.35,-34.12,67.46 |
| 218 | 7 | L-Limbic | PreMot+SuppMot | -7.75, -22.37,46.05 |
| 9 | 6 | R-Prefrontal | AntPFC | 28.88,51.14,18.68 |
| 247 | 6 | L-Celebellum | Cerebellum | -10.29, -81.71, -32.29 |
| 11 | 4 | R-Prefrontal | DlPFC(dorsal) | 37.62,35.39,31.09 |
| 160 | 4 | L-Motor-strip | PreMot+SuppMot | -16.2, -19.23, 69.53 |
| 259 | 4 | L- Basal ganglia | Caudate | -10.4,10.9, -8.13 |
| 191 | 3 | L-Temporal | MiddleTempGyrus | -58.98, -29.96, 3.49 |
| 39 | 3 | R-Parietal | Primsensory | 20.01, -33.24,69.77 |
| 158 | 3 | L-Motor-strip | PrimMotor | -41.59, -14.69, 44.79 |
| 25 | 3 | R-Motor-strip | PreMot+SuppMot | 6.97, -8.07,52.92 |

B. Negative network of akinetic-rigid

| Node Number | Sum of connections | Region | Brodmann's area | MNI |
| --- | --- | --- | --- | --- |
| 159 | 17 | L-Motor-strip | PreMot+SuppMot | -58.09, -5.56, 27.19 |
| 228 | 17 | L-Limbic | Amygdala | -26.79, 2.42, -18.71 |
| 40 | 11 | R-Parietal | PrimSensory | 43.34, -10.81,13.91 |
| 61 | 11 | R-Temporal | PrimAuditory | 59.18, -3.36, 2.74 |
| 38 | 9 | R-Parietal | Primary sensory | 32.42, -19.19, 49.58 |
| 21 | 9 | R-prefrontal | Broca-operc | 55.35, 9.621, 22.22 |
| 265 | 8 | L-brainstem | Brainstem | -4.97, -21.51, -15.83 |
| 171 | 8 | L-Parietal | PrimSensory | -50.47, -23.79, 41.37 |
| 197 | 7 | L-Temporal | SupTempGyrus | -57.05, -14.52, -6.87 |
| 23 | 7 | R-Motor-strip | Primmotor | 57.78, -8.34,27.32 |
| 163 | 6 | L-Motor-strip | PreMot+SuppMot | -56.98, -3.43,6.82 |
| 158 | 5 | L-Motor-strip | PrimMotor | -41.59, -14.69, 44.79 |

C. Positive network of tremor

| Node Number | Sum of connections | Region | Brodmann's area | MNI |
| --- | --- | --- | --- | --- |
| 27 | 30 | R-Motor-strip | PreMot+SuppMot | 49.21, -4.49, 48.12 |
| 262 | 16 | L-Thalamus | Thalamus | -9.59, -25.43, -1.42 |
| 126 | 12 | R-Thalamus | Thalamus | 10.46, -26.75, -2.17 |
| 33 | 11 | R-Motor-strip | PrimSensory | 41.97, -23.38, 53.41 |
| 128 | 10 | R-Thalamus | Thalamus | 4.46, -9.67,5.24 |
| 132 | 9 | R-Brainstem | Brainstem | 6.31, -24.92, -17.47 |
| 38 | 8 | R-Parietal | PrimSensory | 32.42, -19.19, 49.58 |
| 158 | 8 | L-Motor-strip | PrimMotor | -41.59, -14.69, 44.79 |
| 265 | 7 | L-Brainstem | Brainstem | -4.97, -21.51, -15.83 |
| 79 | 6 | R-Occipital | SecVisual | 6.99, -75.69, -2.84 |
| 171 | 6 | L-Parietal | PrimSensory | -50.47, -23.79,41.37 |
| 211 | 6 | L-Occipital | SecVisual | -8.88, -70.65, -1.67 |

D. Negative network of tremor

| Node Number | Sum of connections | Region | Brodmann's area | MNI |
| --- | --- | --- | --- | --- |
| 185 | 19 | L-Temporal | Temporalpole | -38.01,6.07, -37.86 |
| 202 | 14 | L-Temporal | Parahipp | -29.96, -5.77, -40.93 |
| 54 | 13 | L-Temporal | MiddleTempGyrus | -50, -33.82, -0.66 |
| 235 | 10 | L-Limbic | Parahipp | -21.38, -4.06, 29.37 |
| 249 | 9 | L-Cerebellum | Cerebellum | -34.45, -50.33, -53.98 |
| 190 | 8 | L-Temporal | MiddleTempGyrus | -57.62, -6.37, -22.69 |
| 246 | 7 | L-Cerebellum | Cerebellum | -42.57, -63.71, -46.29 |
| 96 | 7 | R-Limbic | Parahipp | 29.31, -19.56, -26.31 |
| 231 | 7 | L-Limbic | Hippocampus | -22.7, -12.76, -17.43 |
| 252 | 7 | L-Cerebellum | Cerebellum | -46.41, -46.77, -42.86 |
| 37 | 6 | R-Insula | Insula | 38.34, -12.45, -1.09 |
| 63 | 6 | R-Temporal | SupTempGyrus | 61.85, -23.77, -2.81 |

TableS5 Overlapping connections between Akinetic-rigid and tremor connectomes

| Node Number | Sum of connections | | Region | Brodmann's area | MNI |
| --- | --- | --- | --- | --- | --- |
|  | Positive | Negative |  |  |  |
| 235 | 0 | 2 | L-Limbic | Parihipp | -21.38, -4.06, -29.37 |
| 159 | 0 | 1 | L-Motor-strip | PreMot+suppMot | -28.09, -5.55,27.19 |
| 54 | 0 | 1 | L-Temporal | MiddleTempGyrus | -50, -33.82, -0.66 |
| 197 | 0 | 1 | L-Temporal | SuperTempGyrus | -57.05, -14.52, -6.87 |
| 163 | 0 | 1 | L-Motor-strip | PreMot+suppMot | -56.98, -3.43,6.82 |
| 111 | 1 | 0 | R-Celebellum | Cerebellum | 11.72, -84.13, -34.65 |
| 33 | 1 | 0 | R-Motor-strip | PrimSensory | 41.97, -23.38, 53.41 |
